# The Cellular Metabolism and Effects of Gold Complexes

**DOI:** 10.1155/MBD.1994.395

**Published:** 1994

**Authors:** Garry G. Graham, G. David Champion, John B. Ziegler

**Affiliations:** 1 School of Physiology and Pharmacology University of New South Wales Sydney 2052 Australia; 2 School of Paediatrics University of New South Wales Sydney 2052 Australia

## Abstract

Leads to the cellular effects of the anti-arthritic gold complexes may come from the determination of their metabolism by target cells and, possibly, cells in the immediate environment of the target cells. Polymorphonuclear leukocytes (PMN) and mononuclear cells (monocytes and lymphocytes) are present in inflamed joints of patients with rheumatoid arthritis and these cells have been widely used in pharmacological studies on the gold complexes. It is suggested that the cellular effects of the gold complexes are mediated by the production of aurocyanide. According to this hypothesis, PMN metabolize small quantities of thiocyanate to cyanide which, in turn, converts gold complexes, such as aurothiomalate, to aurocyanide (dicyanogold(I)) which inhibits the functions of PMN and other cells. There is now considerable evidence for this hypothesis from *in vitro* studies but there is little *in vivo* work to back up the hypothesis. One of the few *in vivo* studies which tested the hypothesis involved the examination of the activity of aurothiomalate in the treatment of polyarthritis in Hooded Wistar rats. Activity of aurothiomalate is only shown in animals which received thiocyanate. Hydrogen cyanide is a constituent of cigarette smoke and the aurocyanide formed by the interaction with gold complexes and inhaled hydrogen cyanide rapidly diffuses into red blood cells. Because of the metabolism of hydrogen cyanide to thiocyanate in the liver, there are higher plasma levels of thiocyanate in smokers than in non-smokers. Smokers may have a greater incidence of side effects than non-smokers but there appears to be little difference in therapeutic response, possibly because there is sufficient thiocyanate in extracellular fluid, even in non-smokers, to support the conversion of gold complexes to aurocyanide. The relationship between the metabolism and effects of the orally active gold complex, auranofin are less clear. Auranofin itself is taken up by cells with the loss of the ligands bound to gold while its inhibitory activity against the oxidative burst of PMN decreases with increasing cell density. For example, the lucigenin-dependent chemiluminescence of 10^6^  PMN/ml is 46 percent of control at 0.5 μM auranofin but only 2.2 percent in 2.10^5^  PMN/ml in the presence of the same concentration of auranofin. A potentially active gold complex is a plasma component which is taken up by red blood cells.


### THE CELLULAR METABOLISM AND EFFECTS OF GOLD COMPLEXES

Garry G. Graham[1], G David Champion[2] and John B. Ziegler[2]

[1] School of Physiology and Pharmacology
[2] School of Paediatrics, University of New South Wales, Sydney 2052, Australia

**Summary** Leads to the cellular effects of the anti-arthritic gold complexes may come from the the determination of their metabolism by target cells and, possibly, cells in the immediate environment of the target cells. Polymorphonuclear leukocytes (PMN) and mononuclear cells (monocytes and lymphocytes) are present in inflamed joints of patients with rheumatoid arthritis and these cells have been widely used in pharmacological studies on the gold complexes. It is suggested that the cellular effects of the gold complexes are mediated by the production of aurocyanide. According to this hypothesis, PMN metabolize small quantities of thiocyanate to cyanide which, in turn, converts gold complexes, such as aurothiomalate, to aurocyanide (dicyanogold(I)) which inhibits the functions of PMN and other cells. There is now considerable evidence for this hypothesis from *in vitro* studies but there is little *in vivo* work to back up the hypothesis. One of the few *in vivo* studies which tested the hypothesis involved the examination of the activity of aurothiomalate in the treatment of polyarthritis in Hooded Wistar rats. Activity of aurothiomalate is only shown in animals which received thiocyanate. Hydrogen cyanide is a constituent of cigarette smoke and the aurocyanide formed by the interaction with gold complexes and inhaled hydrogen cyanide rapidly diffuses into red blood cells. Because of the metabolism of hydrogen cyanide to thiocyanate in the liver, there are higher plasma levels of thiocyanate in smokers than in non-smokers. Smokers may have a greater incidence of side effects than non-smokers but there appears to be little difference in therapeutic response, possibly because there is sufficient thiocyanate in extracellular fluid, even in non-smokers, to support the conversion of gold complexes to aurocyanide. The relationship between the metabolism and effects of the orally active gold complex, auranofin are less clear. Auranofin itself is taken up by cells with the loss of the ligands bound to gold while its inhibitory activity against the oxidative burst of PMN decreases with increasing cell density. For example, the lucigenin-dependent chemiluminescence of $10^6$ PMN/ml is 46 percent of control at 0.5 μM auranofin but only 2.2 percent in $2.10^5$ PMN/ml in the presence of the same concentration of auranofin. A potentially active gold complex is a plasma component which is taken up by red blood cells.

Gold complexes are one of the groups of drugs used in the long term treatment of rheumatoid arthritis. They are problematic drugs because of their potentially serious side effects and limited therapeutic efficacy. Curtailment of their use has been recommended recently [1] but, while the clinical use of the gold complexes is probably decreasing, they are still used in the treatment of rheumatoid arthritis, particularly when the disease is mild but progressive [2]. While the gold complexes are thus minor drugs, determination of their mechanism of action is still worthwhile. More detailed knowledge of their cellular effects could lead to the development of complexes with improved therapeutic indices, to potential sites of action for new anti-rheumatic drugs and, possibly, even to the pathophysiological basis of rheumatoid arthritis. Leads on the biological interactions of complexes of other heavy metals may also be obtained in studies on the gold complexes.

### Clinical activity of the gold complexes

The clinical effects of the gold complexes are difficult to quantitate, because their slow actions necessitate prolonged clinical trials. Such clinical trials are difficult to design, conduct and analyse. Deficiencies in the design and analysis of clinical trials of the gold complexes include small numbers of patients, lack of consideration of type II statistical errors and inadequate discussion of the reasons for discontinuation of treatment. Such problems make it difficult to accept unequivocally the results of many trials on gold complexes, as well as other slowly acting anti-rheumatic drugs.

Placebo treatment always presents both practical and ethical problems in prolonged clinical trials. The often numerous discontinuations both in the gold and placebo treated groups present marked difficulties in the statistical analysis. Comparison of the disease activity in patients completing the clinical trials may not indicate the overall effect of the drugs or placebo. In particular, numerous terminations due to lack of efficacy in patients given placebo may make a placebo-treated group appear to improve when only the clinical state of the patients completing the trial is measured at the end of the study [3].

Despite the problems of the clinical trials of gold complexes, the following conclusions about the actions of the injectable gold complexes can be made [4]:

1. In responsive patients, the beneficial effect increases slowly for at least six months. Side effects, mainly rashes, are often delayed. Extremely serious side effects, such as aplastic anemia are produced occasionally.

2. The mean response is small but an excellent response occurs in 20 to 35 percent of patients and peaks at 6 to 12 months, but is sustained in only about half these patients. Many patients do not improve significantly. Overall, interpatient differences in the therapeutic response are large, but it does appear that the gold complexes are very effective in some patients. This is a major reason for continued research on the gold complexes.

3. Although the gold complexes are often considered to provide long term treatment for rheumatoid arthritis, their use is usually not continued for many years. For aurothiomalate, only about 20 percent of patients continue treatment after 4 years [5]. Termination of treatment is due largely either to side effects or to lack of efficacy. As discussed above, some patients respond initially but subsequently terminate treatment because of decreased effect. Thus, the effect of the gold complexes may be self-limiting.

4. No relationships between the dosage of aurothiomalate and therapeutic response have been established over the commonly used dose ranges of the injectable complexes although the extremes (10 and 150 mg weekly) have not been compared. The higher doses may be more toxic.

5. There is, on average, slight retardation of joint damage.

6. The injectable gold complexes have similar beneficial activity to other slowly active anti-rheumatic agents, although sulfasalazine and methotrexate have a lower incidence of side effects, while methotrexate has a faster onset of action and, at present, is used more widely than the gold complexes.

7. The orally active complex, auranofin, is slightly less efficacious than the injectable complexes but causes fewer serious side effects. The dosage of auranofin is restricted by its purgative activity and this probably limits its efficacy and serious side effects.

8. Therapeutic activity is shown not only in rheumatoid arthritis but the gold complexes may also have activity in the treatment of psoriatic arthritis, asthma and two less common inflammatory diseases, discoid lupus erythematosus and pemphigus.

These phenomena should be considered in studies on the mechanism of action of the gold complexes. For the actions of the gold complexes to be understood at a molecular and cellular level, the spectrum of clinical effects must be explicable.

### Aurocyanide hypothesis

One hypothesis which has been put forward is that the cellular effect of the gold complexes is mediated by aurocyanide [6,7]. This hypothesis arose from studies on the distribution of gold in red blood cells. During treatment with aurothiomalate, gold is found in much higher concentrations in the red blood cells of smokers than in the same cells in non-smokers [8-10]. The reason is that hydrogen cyanide is inhaled by smokers and forms the very stable complex, aurocyanide (see below) which allows the gold to be taken up and bound to red blood cells [11]. Following this finding, we investigated the effect of aurocyanide on the function of white blood cells, reasoning that, if aurocyanide is taken up by red blood cells, it may also be available to other cells and consequently affect their functions.

There are several experimental findings which are consistent with the aurocyanide hypothesis:

1. Cyanide is formed by the activity of myeloperoxidase in PMN [12]. The major source is probably thiocyanate which is oxidized by myeloperoxidase. This enzyme metabolizes at least some of the hydrogen cyanide further to cyanogen chloride [12]. Myeloperoxidase is present in PMN and monocytes, although it is lost as monocytes are transformed into tissue macrophages. Other sources of cyanide, such as glycine, are possible [13] while very different products of the reaction between thiocyanate and myeloperoxidase have also been recorded. Hypothiocyanite ($OSCN^-$) has been identified as a major product of the enzymatic activity of myeloperoxidase acting on thiocyanate [14] and more work is required on the identification of the products of the reaction.

2. Cyanide reacts with aurothiomalate and related polymeric complexes to yield the very stable complex, aurocyanide. There is good chemical evidence for this step in defined chemical systems [15,16]. At ratios of cyanide to Autm of 2:1 or more, aurocyanide is clearly the major product. At lower ratios, other products are formed, particularly the mixed complex, $RS\text{-}Au\text{-}CN^-$, but some aurocyanide is still present [15-18]. Thus, the major reactions are:

$$Au\text{-}SR + HCN \longrightarrow RS\text{-}Au\text{-}CN^- + H^+ \qquad (1)$$

$$RS\text{-}Au\text{-}CN^- + HCN \longrightarrow Au(CN)_2^- + RSH \qquad (2)$$

The mixed complex may disproportionate to yield aurocyanide and the bis thiolate complex:

$$2\,(RS\text{-}Au\text{-}CN^-) \longrightarrow Au(CN)_2^- + Au(SR)_2^- \qquad (3)$$

The bis thiolate complex may be formed from the liberated thiol and the parent polymeric thiol:

$$AuSR + RSH \longrightarrow Au(SR)_2^- + H^+ \qquad (4)$$

3. There are spectroscopic [7] and chromatographic data [19] demonstrating the formation of aurocyanide by activated PMN when thiocyanate and aurothiomalate are present. Like PMN, monocytes contain myeloperoxidase although the levels of the enzyme are lower than in PMN. Thus, monocytes may also convert thiocyanate to hydrogen cyanide and, consequently, lead to the production of aurocyanide. However, the synthesis of aurocyanide in suspensions of purified monocytes has not been examined as yet.

4. Aurocyanide is a common metabolite of aurothiomalate, aurothioglucose and auranofin in patients treated with these complexes [20]. The plasma concentrations are, however, very low, being about 0.1 to 1 percent of the concentrations which inhibit the functions of PMN and lymphocytes. However, the detection of aurocyanide indicates that this complex is available to cells and has some stability under *in vivo* conditions.

5. Aurocyanide inhibits the oxidative burst of PMN which are usually present in very high numbers in the synovial fluid of inflamed joints at all stages of rheumatoid arthritis. A feature of the effect of aurocyanide on PMN is that its effect is delayed (Figure 1). Initially, the oxidative burst is either not altered or slightly accelerated. Consistent with the delayed inhibition, the depolarisation of PMN, a phenomenon associated with the initiation of the burst, is not inhibited [21].

Two hypotheses may be put forward to explain this delayed effect of aurocyanide on the oxidative burst of PMN. The first is that aurocyanide does not inhibit the transduction pathways involved in the initiation of the burst but inhibits processes required for the maintenance of the burst. A second possibility is that aurocyanide is metabolized further by activated PMN to the active

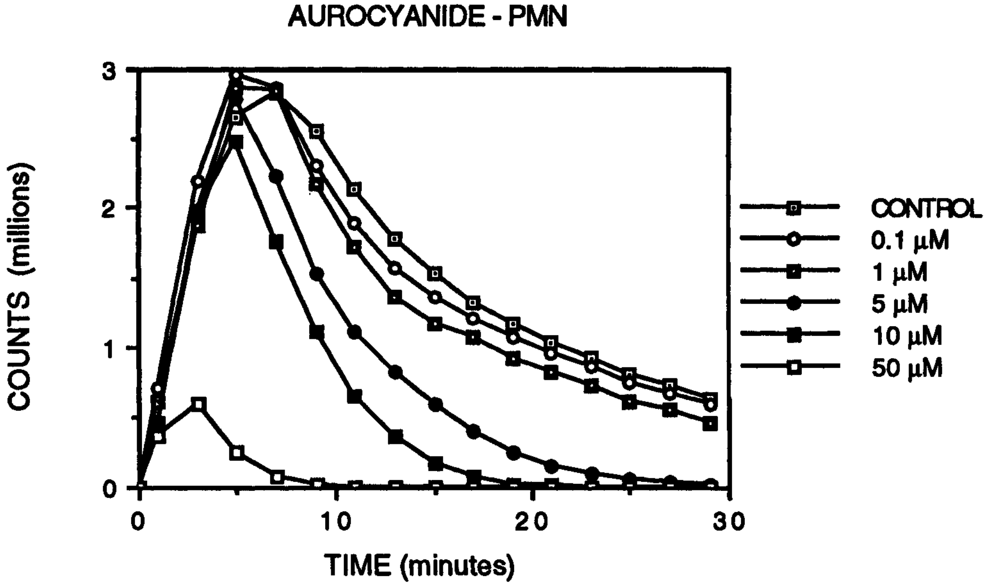

**Figure 1.** The effect of aurocyanide on the production of superoxide (measured as lucigenin-dependent chemiluminescence) by PMN ($2.10^5$/ml). Aurocyanide is active in both the absence and presence of thiocyanate but aurothiomalate is inhibitory only when thiocyanate is present. Data of Rudkowski *et al* [6].

**Table I.** The effect of gold complexes (10 μM) on the production of superoxide (measured as lucigenin-dependent chemiluminescence) by PMN ($10^6$/ml in 50 percent synovial fluid). The concentration of thiocyanate was measured in synovial fluid and the final concentration of thiocyanate was adjusted to 50 μM. The cells were stimulated twice; at time 0 by opsonized zymosan (1 mg/ml), at 30 minutes by phorbol myristate acetate (100ng/ml). Mean values and 95 percent confidence ranges are shown.

| | Chemiluminescence (percent of control) | |
|---|---|---|
| | 0 - 30 minutes | 30 - 60 minutes |
| Aurocyanide | 53 (41-66) | 7 (0 - 16) |
| Aurothiomalate | 71 (43 - 98) | 42 (0 - 85) |
| Auranofin | 93 (78 - 107) | 50 (17 - 84) |

species. As discussed below (point 7), aurocyanide still inhibits the oxidative burst of PMN in the presence of an inhibitor of myeloperoxidase and any activation of aurocyanide probably does not involve myeloperoxidase.

6. Aurothiomalate inhibits the oxidative burst of PMN but only in the presence of physiological concentrations of thiocyanate [7]. Because of the formation of aurocyanide (point 3) and its inhibition of the oxidative burst (point 5), this finding satisfies an important requirement of the aurocyanide hypothesis. A problem in the interpretation of the experiments on the influence of thiocyanate on the activity of aurothiomalate is that thiocyanate itself influences the oxidative burst of PMN. It slightly stimulates the production of superoxide [7], making it difficult to compare the effects of aurothiomalate in the absence and presence of thiocyanate. Could thiocyanate change the response to aurothiomalate because of altered metabolism of PMN, independent of the production of aurocyanide? This question has not been answered but the activity of Autm in the presence of physiological concentrations of thiocyanate and the simultaneous production and activity of aurocyanide provides good evidence for the aurocyanide hypothesis.

The activities of aurocyanide and aurothiomalate have been evaluated in an *in vitro* system which mimics the PMN in inflamed joints *in vivo*. Both gold complexes inhibit the oxidative burst of PMN in 50 percent synovial fluid, with the preformed aurocyanide having the greater effect (Table I). In this system, aurothiomalate tends to be more active than the orally administered complex, auranofin. The conditions of the experiment are critical in demonstrating the activity of aurothiomalate in this *in vitro* system. Aurothiomalate inhibits the oxidative burst of PMN at a cell density of $10^6$/ml in synovial fluid (Table I) but is without activity at $2.10^5$ PMN/ml, presumably because insufficient amounts of hydrogen cyanide and, consequently, aurocyanide are produced at the lower cell density.

7. Sodium azide prevents the inhibitory effect of the thiocyanate/aurothiomalate combination on the oxidative burst of PMN (Table II). Azide is an inhibitor of myeloperoxidase and thus should decrease the conversion of thiocyanate to hydrogen cyanide and, consequently, the formation of aurocyanide and its effect on the oxidative burst of PMN. The effect of azide on thiocyanate/aurothiomalate is therefore consistent with the aurocyanide hypothesis. These results are again somewhat difficult to interpret since azide itself has a late inhibitory effect on lucigenin-dependent chemiluminescence and this experiment requires confirmation using an inhibitor with less influence on the baseline measurement of the oxidative burst. Furthermore, in order to conclude that

**Table II.** The influence of azide on the production of superoxide (measured as lucigenin-dependent chemiluminescence) by PMN ($2.10^5$/ml) in the presence of the aurothiomalate (10 μM) and (10μM/thiocyanate (40μM). Mean values and 95 percent confidence ranges are shown. Control chemiluminescence was measured in the presence of thiocyanate (40 μM) or thiocyanate + azide (10 μM). The cells were stimulated twice; at time 0 by opsonized zymosan (1 mg/ml), at 30 minutes by phorbol myristate acetate (100ng/ml).

| | Chemiluminescence (percent of control) | |
|---|---|---|
| | 0 - 30 minutes | 30 - 60 minutes |
| Aurothiomalate + Thiocyanate | 82 (66 - 99) | 20 (0 - 41) |
| Aurothiomalate + Thiocyanate + Azide | 101 (81 - 122) | 83 (58 - 108) |

myeloperoxidase is responsible for the production of hydrogen cyanide and hence lead to the production of aurocyanide, it must also be shown that azide does not influence the inhibitory effect of pre-formed aurocyanide on the oxidative burst of PMN. As discussed above (point 5), an inhibitory effect of aurocyanide is still seen in the presence of azide although, as yet, only limited experiments have been conducted.

Interestingly, aurocyanide is oxidized by hypochlorite, the major product of myeloperoxidase (see paper by Shaw et al in this volume). The observation that azide does not prevent the inhibitory actions of aurocyanide on the oxidative burst of PMN indicates that the oxidation of aurocyanide is not a prerequisite for its effects on the oxidative burst of PMN. However, such oxidation may limit the net availability of aurocyanide when it is produced by PMN and its further metabolism by PMN is an aspect of the biological interactions of aurocyanide which requires further examination.

8. Aurocyanide is an anti-arthritic agent in rats [23]. Aurocyanide (10 μmol/kg) inhibits the development of experimental polyarthritis in two strains of rats (Dark Agouti and Hooded Wistar) while potassium cyanide (10 μmol/kg) is inactive. In one of these strains (Dark Agouti), aurothiomalate (30 μmol/kg) also suppresses the development of the arthritis while this gold complex is inactive in the other strain (Hooded Wistar). In the latter strain, the aurothiomalate is active when thiocyanate (25 mM) is included in the drinking water. This result may be interpreted as indicating that aurothiomalate is activated *in vivo* when high levels of thiocyanate are available to support the production of aurocyanide which has anti-arthritic activity. Thus, this result is consistent with the aurocyanide hypothesis. However, the administration of thiocyanate exacerbates the polyarthritis in rats and other explanations may be possible [23].

Interestingly, the anti-arthritic activity of silver thiomalate and potassium silver cyanide largely parallels the activity of aurothiomalate and aurocyanide. Both silver thiomalate and potassium silver cyanide are active the Dark Agouti rats but silver thiomalate is only active in the Hooded Wistar rats if they also ingest thiocyanate [23].

9. Aurocyanide is an inhibitor of the proliferation of lymphocytes. Because of the potential involvement of lymphocytes in the pathogenesis of rheumatoid arthritis, the effects of aurocyanide on these cells are of particular interest. Aurocyanide is a potent inhibitor of the mitogen-induced proliferation of lymphocytes, with an EC50 of about 1 μM in cultures of mixed mononuclear cells, well below the plasma concentrations of total gold during therapy with aurothiomalate. Plasma proteins bind aurocyanide and decrease its pharmacological activity but, while the cultures of mixed mononuclear cells contain foetal calf serum, aurocyanide still potently suppresses the proliferation of lymphocytes. The approximate EC50 (1μM) is about 10 percent of the usual concentrations of total gold in plasma during treatment with aurothiomalate.

A feature of the effect of aurocyanide on the proliferation of lymphocytes is that its effect increases when added late in the incubations. This is shown particularly at 0.5 μM aurocyanide. At this concentration, aurocyanide is without significant effect when added at the commencement of the incubations but there is considerable effect when added at 1 and 2 days after the commencement of the 3 day incubations (Table III). This result indicates that aurocyanide has a direct effect on the lymphocytes and not on the monocytes which are required for the activation of lymphocytes. Interestingly, tolerance to aurocyanide appears to develop. Thus, when aurocyanide is added twice; at the commencement of the incubations and at 2 days, the inhibitory effect of aurocyanide is less than that seen when added only once at 2 days (Table III), even though the total concentration of added aurocyanide is now 1 μM.

While aurocyanide inhibits the proliferation of lymphocytes, similar inhibition will only occur *in vivo* if the lymphocytes are exposed to sufficient aurocyanide. Infiltrations of lymphocytes are commonly found in the synovium of joints of patients with rheumatoid arthritis while large numbers of PMN are present in synovial fluid. It is probable that the PMN in synovial fluid are the major cells leading to the production of aurocyanide and the question is whether the aurocyanide formed by the PMN is available to the lymphocytes at some distance from the synovial fluid. The concentration of the complex formed by $10^6$ PMN/ml *in vitro* is is in the range 0.5 to 1 μM [19], while the concentrations in plasma are about two to three orders of magnitude lower. It is probable that the concentrations of aurocyanide in the synovium are higher than in plasma but the concentrations of aurocyanide available to lymphocytes are unknown. An answer to this question may be critical to the acceptance of the aurocyanide hypothesis since it is generally considered that these cells are a major target of anti-arthritic drugs.

**Table III.** The effect of 0.5 μM aurocyanide on the proliferation of lymphocytes when added at various times to cultures of mixed mononuclear cells. The proliferation of the lymphocytes was measured by the incorporation of $^{14}C$-thymidine added at 2 days after the commencement of the 3 day cultures. Mean values and 95 percent confidence ranges are shown.

| Time of addition of aurocyanide | Incorporation of thymidine (percent of control) |
|---|---|
| 0 | 95 (63 - 127) |
| 1 hour | 92 (61 - 123) |
| 4 hours | 97 (65 - 128) |
| 1 day | 58 (38 - 78) |
| 2 days | 29 (11 - 47) |
| 0 + 2 days | 62 (46 - 78) |

Like aurocyanide, aurothiomalate is an inhibitor of the proliferation of lymphocytes in suspensions of mixed mononuclear cells. Aurothiomalate is, however, much less potent than aurocyanide. When added from the commencement of incubations, the EC50 of aurothiomalate is approximately 100 μM [22], making aurothiomalate about two orders of magnitude less potent than aurocyanide.

The activity of aurothiomalate is not only much less potent than aurocyanide, but the effect of the two complexes is markedly different when added late in the incubations. Thus, in contrast to aurocyanide, the inhibitory effect of aurothiomalate decreases when added at later times after the commencement of the incubation [22] as we have confirmed. This and related observations led Lipsky and Ziff [22] to suggest that the effect of aurothiomalate was on monocytes which act as accessory cells in the early stages in the activation of the proliferation of lymphocytes. However, we have found recently that aurothiomalate does not inhibit the proliferation of lymphocytes if washed off the mononuclear cells at times up to 24 hours after the commencement of the incubations. Thus, aurothiomalate may require continued exposure to lymphocytes to inhibit their proliferation.

### Clinical significance of the formation of aurocyanide

At this stage, it is only possible to speculate on the significance of the formation of aurocyanide *in vivo*. However, if the effects of the gold complexes are indeed mediated by aurocyanide, then reasons for some clinical responses to the anti-arthritic gold complexes may be suggested.

Apart from the slow clinical response to the gold complexes, the main feature of the clinical response to their use is the interpatient variation in both the therapeutic response and the development of side effects. The formation of aurocyanide may very well depend upon the numbers and level of activation of PMN and monocytes in joints and the access of aurocyanide to their target cells. As discussed above, PMN and monocytes may be responsible for the formation of aurocyanide, but may not be the ultimate target cell. Interpatient variation in the synthesis and delivery of aurocyanide to target cells could easily lead to the variable clinical response.

Clinical responses to the gold complexes indicate that their anti-rheumatic activity may decrease with continued treatment. Factors associated with the production of aurocyanide may lead to tolerance. According to the aurocyanide hypothesis, the formation of aurocyanide is dependent upon the activity of PMN or monocytes. If the activity of these cells subsides to a low level, the production of aurocyanide may be insufficient to produce a marked suppression of the inflammation of rheumatoid arthritis. However, the disease may continue at a low level although still sufficient to lead to joint damage. Our introductory work on the proliferation of lymphocytes indicates that resistance to aurocyanide may occur, also consistent with tolerance to gold complexes.

One of the most significant side effects of the gold complexes is the development of ulceration of mucous membranes of the mouth. Could this be related to the formation of aurocyanide? High levels of both peroxidase and a substrate, thiocyanate, are present in saliva. The subsequent production of hydrogen cyanide [25] and aurocyanide may lead to this production of mouth ulcers.

Interest in the medical activity of aurocyanide first arose just over 100 years ago when the great German microbiologist, Robert Koch, found that aurocyanide inhibited the growth of *mycobacterium tuberculosis* [26]. Aurocyanide was, however, poorly tolerated and was reported to be ineffective in the treatment of tuberculosis. Possibly its lack of activity *in vivo* was due to uptake by red blood cells and binding to plasma proteins. Other gold complexes were subsequently introduced for the treatment of tuberculosis, also without marked benefit, but were eventually found to be useful in the treatment of rheumatoid arthritis.

### Smoking and the clinical response to gold

One of the uncertainties about the aurocyanide hypothesis concerns the lack of any clear effect of smoking on the clinical response to aurothiomalate. Smokers inhale hydrogen cyanide which is metabolized to thiocyanate in the liver. Consequently, smokers have higher plasma concentrations of thiocyanate than non-smokers and, since thiocyanate is metabolized back to hydrogen cyanide in PMN, a higher availability of cyanide is anticipated in smokers, both because of its direct inhalation and also its greater production by PMN. Thus, greater synthesis of aurocyanide is anticipated in smokers. However, the therapeutic response to aurothiomalate is not altered by smoking [10]. Terminations to treatment with aurothiomalate were also not related to smoking [5], although a greater [24] or earlier incidence [8] of side effects has been reported in smokers. While smoking may influence the side effects to the gold complexes, any effect is of degree only, since smokers do have side effects to their dosage with the gold complexes.

The *in vitro* data on the effects of the aurothiomalate/thiocyanate on PMN (point 6 above) indicate that levels of thiocyanate in non-smokers causes the activation of aurothiomalate in these cells. Possibly, the levels of thiocyanate in non-smokers are high enough to allow the production of sufficient aurocyanide to mediate the effects of aurothiomalate. Other factors, such as the degree of activation and the numbers of PMN at sites of inflamation, may be responsible the marked interpatient differences in therapeutic effects. The determination of the effect of a range of concentrations of thiocyanate on the PMN-mediated production of aurocyanide from aurothiomalate and the subsequent effect of the aurocyanide on the oxidative burst of PMN should help to answer this question.

The interaction between smoking and the clinical effects of the gold complexes is confounded by a possible relationship between smoking and the development of rheumatoid arthritis. While smoking does not appear to be a major factor, it may increase the incidence of rheumatoid arthritis in young males [27] who rarely suffer from the disease.

### Auranofin

The active form of gold during treatment with auranofin has not been determined. Leads may again come from studies on the distribution of gold in red blood cells. During treatment of rheumatoid patients with auranofin, gold is present in red blood cells whether the patients are smokers or not [10]. Smoking does, however, increase the levels of gold in red blood cells although not to nearly the same extent as seen during aurothiomalate therapy [10]. Thus, there may be some interaction with hydrogen cyanide and auranofin or its metabolites *in vivo*.

Following the administration of auranofin to man [10] and rats [28], plasma contains a gold complex which is transported into red blood cells. This was demonstrated by administering auranofin orally, sampling blood and then incubating the plasma with fresh red blood cells. Interestingly, this mobile gold complex may exist only for a limited time after the oral administration of auranofin. When plasma was collected 48 hours after dosage of auranofin to rats, some of the gold present diffused into red blood cells but, by contrast, when plasma was collected 72 hours after dosage with auranofin, gold was still present but none was transported into fresh red blood cells [28]. This mobile form of gold has never been identified. If this complex can diffuse or be transported into red blood cells, it could be taken up by target cells. This mobile form of gold is probably not auranofin since the parent complex readily binds to plasma albumin and probably does not last as long as 48 hours in the circulation.

Apart from these limited studies with blood, studies on the uptake as well as the cellular effects of auranofin have been conducted with auranofin itself. The cellular uptake has been most clearly studied with a macrophage-like cell line, RAW 264.7 cells. The cellular uptake was studied using auranofin labelled in three positions: gold, the triethylphosphine ligand and the tetraacetylthioglucose ligand. The amount of gold taken up was much greater than the amount of triethylphosphine and very much greater than that of the thiol ligand, tetraacetylthioglucose [29]. This experiment indicated that, not only was the gold concentrated in the cells, but that the gold was separated from its ligands.

The strong association between the gold and cells indicates that the cellular effects of auranofin may be dependent upon the numbers of cells in a cellular system as well as the concentration of the auranofin. Thus, the amount of gold per cell should decrease with increasing cell densities, with the result that the cellular effects of auranofin should decrease with increasing cell density. This prediction has now been observed experimentally (Table IV). It is difficult to compare quantitatively the degree of inhibition at the two densities of PMN tested, because the fractional inhibition of the oxidative burst does not follow simple dose response relationships and the semi-logarithmic plots of fractional inhibition versus concentration of auranofin are not parallel. The inhibitory effect of auranofin is, however, generally greater at the lower concentration of auranofin (Table IV). No such relationship between cell density and the activity of aurocyanide has been detected [6].

**Table IV.** Relationship between the concentration of auranofin and the depression of the production of superoxide (measured as lucigenin-dependent chemiluminescence) at two cell densities of PMN. Mean values and 95 percent confidence ranges are shown.

| Auranofin concentration (μM) | Chemiluminescence (Percent of control) $2.10^5$ PMN/ml | $10^6$ PMN/ml |
|---|---|---|
| 0.01 | 89 (66 - 113) | 101 (93 - 108) |
| 0.02 | 95 (75 - 115) | 77 (66 - 88) |
| 0.05 | 55 (39 - 70) | 86 (76 - 97) |
| 0.1 | 54 (32 - 75) | 74 (64 - 83) |
| 0.2 | 18 (0 - 36) | 67 (49 - 85) |
| 0.5 | 2.2 (1.6 - 2.8) | 46 (29 - 63) |
| 1 | 2.3 (1.3 - 3.3) | 27 (18 - 37) |
| 2 | 1.6 (1.2 - 2.0) | 5 (0 - 10) |

The action of auranofin on the oxidative burst of PMN is quite complex with at least a dual interaction. At low concentrations ($2.10^5$ PMN/ml + 0.5 μM auranofin), the effect of this complex resembles those of aurocyanide. Both show a late inhibition of the oxidative burst of PMN (Table IV) without inhibiting the depolarisation of the cells. At higher concentrations, auranofin decreases the burst from its initiation and, consistent with an early effect on the oxidative burst, also inhibits the depolarisation of the PMN [21].

A notable activity of auranofin is its high potency in inhibiting the growth of cells from progenitor cells in human bone marrow. Auranofin inhibits this *in vitro* myelopoiesis at concentrations ranging from $10^{-9}$ to $10^{-7}$ M [30]. The potent effect of auranofin may be related to the low inoculum of cells ($5.10^4$) and the consequent high uptake of gold per cell.

Overall, the cellular uptake and activity of auranofin has been studied in considerable detail but the relevance of this work to its clinical efficacy is, however, not clear since the work requires confirmation using metabolites which are present in plasma or at sites of rheumatoid disease.

### References

1. Epstein WV. *Journal of Rheumatology* 1989; **16**: 1291-1294.
2. Cash JM, Klipel JH. *New England Journal of Medicine* 1994; **330**: 1368-1375.
3. Williams HJ, Ward JR, Dahl SL, *et al. Arthritis and Rheumatism* 1988; **31**: 702-713.
4. Champion, GD, Graham GG, Ziegler JB. *Bailliere's Clinical Rheumatology* 1990; **4**: 491-534.
5. Sambrook PN, Browne CD, Champion GD, *et al. Journal of Rheumatology* 1982; **9**: 932-934.
6. Rudkowski R, Graham GG, Champion GD, Ziegler JB. *Biochemical Pharmacology* 1990; **39**: 1687-1695.
7. Graham GG, Dale MM. *Biochemical Pharmacology* 1990; **39**: 1697-1702.
8. Graham GG, Haavisto TM, McNaught PJ,*et al. Journal of Rheumatology* 1982; **9**: 527-531.
9. James DW, Ludvigsen NW, Cleland LG, Milazzo SC. *Journal of Rheumatology* 1982; **9**: 532-535.
10. Lewis D, Capell HA, McNeil CJ, *et al. Annals of the Rheumatic Diseases* 1983; **42**: 566-570.
11. Graham GG, Haavisto TM, Jones HM, Champion GD. *Biochemical Pharmacology* 1984; **33**: 1257-1262.
12. Stelmaszynska T. *International Journal of Biochemistry* 1986; **18**: 1107-1114.
13. Stelmaszynska T. *International Journal of Biochemistry* 1985; **17**: 373-379.
14. Thomas EL, Fishman M. *Journal of Biological Chemistry* 1986; **261**: 9694-9702.
15. Graham GG, Bales JR, Grootveld MC, Sadler PJ. *Journal of Inorganic Biochemistry* 1985; **25**: 163-173.
16. Lewis G, Shaw CJ. *Inorganic Chemistry* 1986; **25**: 58-62.
17. Isab AA. *Journal of Inorganic Biochemistry* 1992; **46**; 145-151.
18. Hormann-Arendt AL, Shaw CF. *Inorganic Chemistry* 1990, **29**: 4683-4687.
19. Graham GG, Champion GD, Haddad PR, *et al. Australian and New Zealand Journal of Medicine* 20: 505 (1990).
20. Elder RC, Zhao Z, Zhang Y, *et al. Journal of Rheumatology* 1993; **20**: 268-272.
21. Rudkowski R, Ziegler, Graham GG, Joulianos G. *Biochemical Pharmacology* 1992; **44**: 1091-1098.
22. Lipsky PE, Ziff M. *Journal of Clinical Investigation* 1977; **59**: 455-466.
23. Whitehouse MW, Vernon-Roberts B. *Australian and New Zealand Journal of Medicine* 1993; **23**: 555.
24. Kay EA, Jayson MIV. *Scandinavian Journal of Rheumatology* 1987; **16**: 241-245.
25. Lundquist P, Rosling H, Sorbo B. *Arch Toxicol* 1988; **61**: 270-274.
26. Koch R. *Deutsche Medicinische Wochenschrift* **16**: 171-173.
27. Heliovaara M, Aho K, Aromaa A, *et al. Journal of Rheumatology* 1993; **20**: 1830-1835.
28. Walz DT, DiMartino MJ, Griswold DE. *Rheumatology* 1983; **8**: 99-117.
29. Snyder RM, Mirabelli CK, Crooke ST. *Biochemical Pharmacology* 1986; **35**: 923-932.
30. Hamilton JA, Williams N. *Journal of Rheumatology* 1987; **14**: 216-220.

